# Primary pigmented meningeal melanocytoma originating in Meckel cave in a patient with carney complex: A case report

**DOI:** 10.1097/MD.0000000000018783

**Published:** 2020-01-17

**Authors:** Abdulrahman Hamad Al-Abdulwahhab, AbdulAziz Mohammad Al-Sharydah, Sari Saleh Al-Suhibani, Hadeel Al-Shayji, Ibtihal Al-Saad, Wissam Al-Issawi

**Affiliations:** a Diagnostic and Interventional Radiology Department; b Neurosurgery Department, Imam Abdulrahman Bin Faisal University, King Fahd Hospital of the University, Saudi Arabia.

**Keywords:** Carney complex, computed tomography, magnetic resonance imaging, melanocytoma, sickle cell disease

## Abstract

**Rationale:**

Primary melanin-producing tumors are rare extra-axial neoplasms OPEN of the central nervous system. In the literature, few case reports have discussed neoplasms involving the cavernous sinus; of these, only 4 have reported on neoplasms originating in Meckel cave. The diagnostic approach, including clinical and radiological analysis, is challenging, and cytopathological assessment with a molecular basis is the best approach to discriminate between these lesions. Herein, we discuss the pathophysiology, diagnostic approach, intraoperative features, and postoperative management in a unique case of primary pigmented meningeal melanocytoma originating in Meckel cave in a patient who was diagnosed with Carney complex (CCx) and sickle cell disease (SCD).

**Patient concerns:**

A 23-year-old man diagnosed with SCD had also been diagnosed previously with CCx, without any familial history or neurocutaneous melanosis. He had experienced headaches accompanied by left facial pain and paresthesia for 2 months.

**Diagnosis:**

The initial computed tomography scan and magnetic resonance imaging (MRI) revealed a mass arising from the left Meckel cave. On MRI, it followed the signal intensity of melanin. He underwent subtotal resection of the mass. Considering the patient's history of CCx, melanocytic schwannoma was the most relevant diagnosis. A postoperative histopathological examination was suggestive of benign pigmented meningeal melanocytoma.

**Interventions:**

The patient underwent an uneventful subtotal resection of the mass through a left temporal linear incision.

**Outcomes:**

The patient showed progressive improvement of neurologic deficits, and after 2 years of follow-up, he did not present with any new complaints.

**Lessons:**

To the best of our knowledge, this is the first report of the unusual presentation of both SCD, as well as of primary pigmented meningeal melanocytoma in a patient with CCx. Complete surgical resection can be curative in most cases of melanocytoma. The presence of CCx with SCD suggests potential shared genetic contributions that will require further exploration.

## 1 Introduction

Primary melanocytic tumors of the central nervous system are extremely rare neuroectodermal neoplasms arising from the leptomeninges.^[1]^ They commonly occur in the fourth and fifth decade of life and are very rare in children and adolescents.^[2]^ The annual incidence of these lesions has been estimated to be approximately 1 per 10 million people, with a predilection for women.^[1]^ They commonly involve the posterior fossa and upper cervical spine due to the presence of melanocytes in these sites.^[2]^

According to the World Health Organization classification of primary brain tumors, meningeal melanocytomas are classified under a subgroup of primary melanocytic neoplasms that have intermediate grade properties with low mitotic activity not fulfilling the criterion for malignant melanoma.^[3]^ They either manifest focally, as is the case for melanocytoma or primary malignant melanoma, or in a diffuse manner, as is observed in cases of leptomeningeal melanocytosis and primary leptomeningeal melanomatosis.^[4]^

Only 8 patients have been reported with primary melanocytoma originating from the cavernous sinus, 4 of whom presented with melanocytoma originating specifically from Meckel cave.^[2,5–11]^ Herein, we discuss the pathophysiology, diagnostic approach, intraoperative features, and postoperative management in a unique case of primary pigmented meningeal melanocytoma originating in Meckel cave in a patient who was diagnosed with Carney complex (CCx) and sickle cell disease (SCD).

### 1.1 Patient information

**Figure 1:**
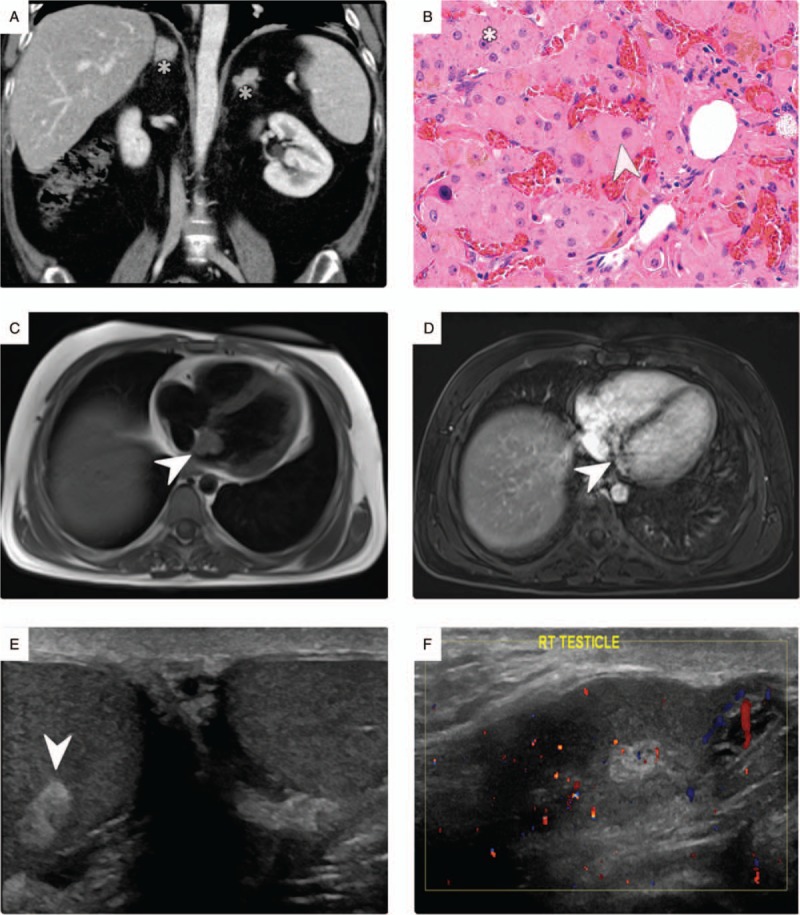
Carney complex associations. (A) Contrast-enhanced coronal computed tomography scan of the abdomen in portal venous phase and soft tissue window demonstrate bilateral uncalcified adrenal masses (asterisks). (B) Postsurgical microscopic histopathological examination reveals sharply circumscribed, unencapsulated nodules (arrowhead) within the adrenal cortex, composed of large eosinophilic lipid-poor cells similar to the zona reticularis. The cells have enlarged pleomorphic nuclei (asterisk), prominent nucleoli, and lipofuscin deposits. No evidence of necrosis or increased mitotic activity are observed; these findings are consistent with primary pigmented nodular adrenocortical disease. (C, D) Cardiac magnetic resonance imaging with and without contrast show a small enhancing lesion within the left atrium appearing to be cardiac myxoma (arrowheads). (E, F) Grayscale and color Doppler ultrasound of the scrotum in glass and longitudinal views show an echogenic hypovascular mass in the right testis (arrowhead), with multiple foci of calcifications that showed stability over time.

A 20-year-old male patient with a known case of SCD was diagnosed with CCx at our hospital when he was admitted with hypertension. During his first visit to the hospital, his work-up analysis revealed bilateral primary pigmented nodular adrenocortical disease, cardiac myxoma, and calcified testicular tumor (Fig. 1). He revisited our hospital 3 years later in June 2016, complaining of frequent headaches accompanied by left facial pain and paresthesia for the past 2 months.

### 1.2 Clinical findings

The patient's symptoms gradually worsened, especially with chewing and exposure to the cold. Neurological examination revealed left fifth and sixth cranial nerve palsies. There was no history of neurocutaneous melanosis or CCx in his family.

### 1.3 Diagnostic assessment

**Figure 2:**
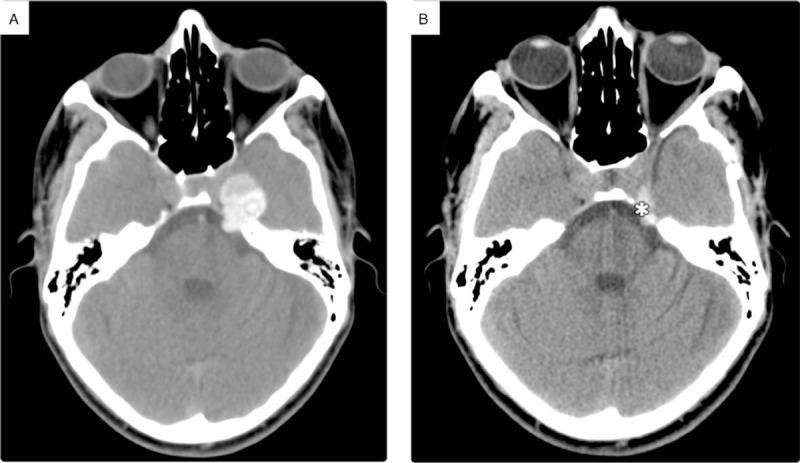
Unenhanced axial computed tomography scans of the brain. (A) Heterogeneously hyperdense mass lesion occupying the left Meckel cave inducing mass effect along the ipsilateral lateral cavernous sinus and extending to a cisternal portion of the trigeminal nerve. (B) Follow-up post subtotal resection through left temporal craniotomy with the small residual lesion (asterisk).

**Figure 3:**
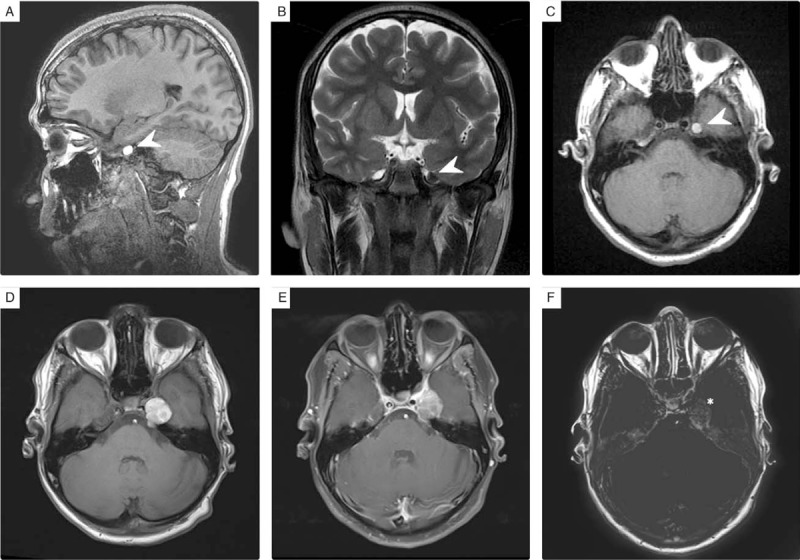
Multisequential multiplanar magnetic resonance images of the brain with and without contrast. Well-defined extra-axial mass (arrowhead), arising from the left Meckel cave, showing bright T1 and dark T2 signal intensities (A–C). Interval increased maximum axial dimension of the mass at 1-year follow-up imaging (D–F). The subtracted image between post-contrast (E) and pre-contrast T1 (D) exhibits a faint peripheral rim of enhancement (asterisks) along the postero-lateral margin of the mass (F).

An initial brain computed tomography (CT) scan revealed a well-defined mass that originated from the left Meckel cave. Its internal texture appeared heterogeneously hyperdense on an unenhanced CT scan (Fig. 2A). Cranial magnetic resonance imaging (MRI) further characterized the mass, presenting with a bright signal on T1-weighted images and very dark signal on T2-weighted images in accordance with the intensity of melanin. It showed a faint peripheral enhancement on the subtracted T1 post-contrast imaging (Fig. 3). The most relevant neuroimaging diagnosis was melanotic schwannoma as part of the CCx; other differential diagnoses included melanocytoma and melanoma. The maximum axial dimension of the mass increased from 0.4 cm in June 2016 to 1.9 cm in June 2017; this led to compression of the cisternal portion of the trigeminal nerve, thus explaining the patient's symptoms.

### 1.4 Therapeutic intervention

**Figure 4:**
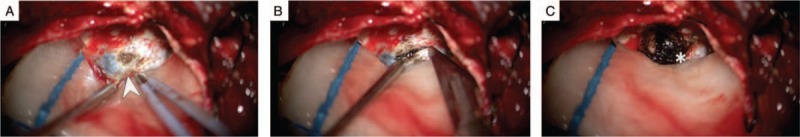
Intra-operative images. (A) The covering dura above the bulge of the mass (arrowhead) is exposed, (B) revealing a coal-black encapsulated mass (asterisk) originating from Meckel cave (C).

Subsequently, subtotal resection of the mass was performed through a left temporal linear incision and by creating a burr hole above the zygoma, followed by craniotomy. The dura opened in a “C” shape, and the dura above the bulging mass was exposed; this revealed a coal-black, encapsulated mass, originating in Meckel cave and adhering tightly to the trigeminal nerve. The tumor was removed in pieces until the vessels were apparent and a subtotal tumor resection was achieved. Grossly, the tumor was a soft, fragile, pigmented mass covered with a thick white capsule (Fig. 4).

Microscopically, the tumor revealed nests of epithelioid-cell infiltration and spindle-shaped neoplastic cells with heavy melanin pigmentation. The cells contained typical macro-nuclei with few irregularities in the nuclear contours and prominent nucleoli. Nevertheless, the cytoplasm was abundant, and neither mitosis nor necrosis was observed.

The tumor was histopathologically examined for cell markers, including cluster of differentiation (CD)136, CD34, and KI67 protein. The tumor was also tested for immunoreactivity staining with epithelial membrane antigen, human melanoma black 45 (HMB45), microphthalmia-associated transcription factor, tyrosinase, vimentin, S100 protein, melanoma antigen (Melan-A), and reticulin. The results were difficult to interpret because of the heavy pigmentation; however, a positive expression of Melan-A, S-100, and HMB45 was noted. Significantly, there was total depletion of reticulin and an absence of Psammoma bodies. Other immunohistochemical findings were predominantly negative and cellular proliferation, measured by staining for Ki-67, was <1%.

**Figure 5:**
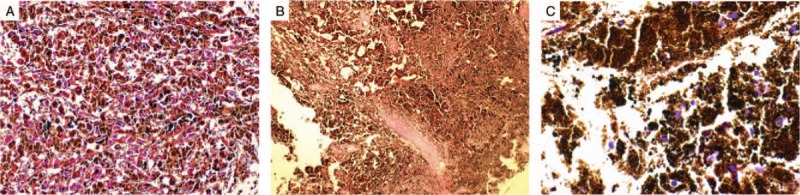
Cytopathological studies of tissue sections. (A) Low-power photomicrograph (hematoxylin and eosin, ×200) shows heavily pigmented lesion composed of polygonal cells with deposition of melanin pigment in the cytoplasm arranged in fascicles with abundant extracellular melanin deposits. Positive immunoreactivity to HMB45 (B) and Melan-A (C), which are antigens present in melanocytic tumors. No psammomatous calcifications or cytological atypia were seen with depletion of reticulin. These findings confirm the diagnosis of melanocytoma.

However, based on these findings, a diagnosis of meningeal melanocytoma rather than melanocytic schwannoma or malignant melanoma was established (Fig. 5).

### 1.5 Follow-up and outcomes

The patient's postoperative course was uneventful as observed through continuous follow-up imaging (Fig. 2B) and progressive improvement of the neurologic deficits. The patient was discharged after postoperative monitoring for 2 weeks, during which time no neurological symptoms were observed; he was followed-up regularly (every month for the first 6 months following discharge and every 3 months thereafter) at the neurosurgery clinic for 2 years and did not present with any new complaints.

## 2 Discussion

To the best of our knowledge, there are only 4 cases of primary meningeal melanocytoma originating in Meckel cave described in the literature (Table 1). Of interest, none of these were associated with SCD. Primary melanocytic tumors of the central nervous system arise from the leptomeningeal melanocytes, with a spectrum ranging from well-differentiated benign meningeal melanocytomas to malignant melanomas. However, atypical melanocytic melanocytomas, which have clinical and pathological characteristics between benign melanocytomas and malignant melanomas, are considered borderline tumors.^[2]^

**Table 1:**
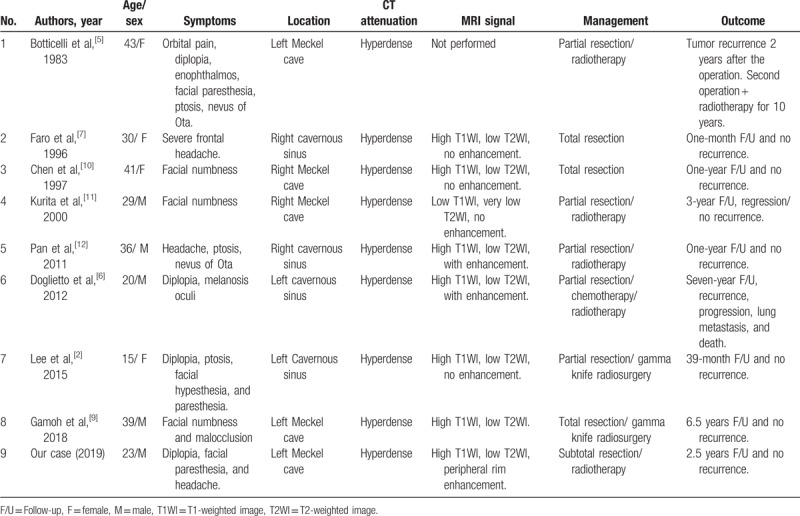
Summary of reported cavernous sinus meningeal melanocytoma cases.

These lesions typically appear on CT scans as extra-axial isodense to hyperdense lesions, with dural and meningeal attachment. Further, they may show homogeneous enhancement after contrast administration.^[6]^ On MRI scans, they usually present with homogeneous bright intensity on T1-weighted images and dark signal intensity on T2-weighted images because of the paramagnetic effects of free melanin radicals. Besides, they show homogeneous enhancement after gadolinium contrast administration.^[2]^ However, this signal pattern on MRI scans may vary based on the melanin and hemorrhagic concentration within the lesion; the lesion can present a heterogeneous signal intensity and a variable contrast-enhancing pattern.^[6]^

Our case showed the typical CT and MRI appearance, which informed the provisional diagnosis of the lesion as melanocytic schwannoma, particularly in the context of all other findings and the presentation of CCx, which represents a spectrum of associated pathologies.^[13]^ Although melanocytomas reportedly have imaging features similar to those of other dural tumors,^[6]^ after gadolinium contrast administration, the lesion in our case showed heterogeneous enhancement; in addition, after performing a subtraction of T1 post-contrast from conventional T1 pre-contrast sequence, a faint peripheral posterolateral enhancement was observed (Fig. 3). Interestingly, this was not reported in other cases in the literature (Table 1).

Histopathological analyses have played a significant role in the diagnosis of meningeal melanocytoma and have helped to rule out similar pathological conditions with similar characteristics.^[2]^ Microscopically, the majority of meningeal melanocytoma cases present as well-circumscribed, non-encapsulated lesions, consisting of several nests or fascicles of epithelioid and spindle cells with little nuclear atypia and abundant melanin pigmentation. Further, immunohistochemical diagnostic criteria require S-100 positivity, expression of Melan-A and/or HMB-45, as well as a nested positive staining pattern in the basement membrane with negative staining to the epithelial membrane antigen and Psammoma bodies.^[14]^

Until recently, distinguishing between melanocytoma and melanotic schwannoma has been challenging, particularly with small specimens. However, melanocytic schwannoma shows abundant deposition of reticulin material in the basement membrane around individual tumor cells; such deposition is usually absent in melanocytic tumors.^[15]^ In our case, we observed abundant extracellular melanin deposition with little nuclear atypia and no Psammoma bodies (Fig. 5). Therefore, the diagnosis of melanocytoma was favored owing to the depletion of reticulin and absence of Psammoma bodies. Other diagnostic approaches include the recent molecular study of GNAQ gene mutations, which permit highly accurate discrimination between leptomeningeal melanocytic lesions and melanotic schwannoma.^[16]^

The survival rate among patients with an untreated meningeal melanocytoma in the central nervous system is 5 to 10 years.^[2,17]^ Among patients who undergo complete surgical resection or an incomplete resection with local radiation therapy, the survival rate might reach 100%; this percentage can drop to 46% in cases of incomplete resection.^[17]^ Thus, we believe that the optimal strategy for managing melanocytomas is complete resection; in case complete resection is impossible, as much of the tumor should be resected as possible, followed by postoperative local radiotherapy.

In our case, complete resection was inadvisable as the lesion was situated in a sensitive area with a high chance of vascular injury. The importance of post-resection radiotherapy depends on the degree of meningeal melanocytoma proliferation, which is controlled by Ki-67, a cellular marker that determines the growth fraction of the cell population.^[17]^ In our case, Ki-67 was very low (<1%). We therefore believed that the chances of recurrence or malignant transformation were low in our patient. Regardless, the possibility of local recurrence, regrowth, or malignant transformation should be considered, and follow-up neuroimaging is advised.^[18]^ This may help the referring clinicians to understand the progression of the disease and inform treatment decisions.^[15]^ In our case, follow-up imaging over 2 years showed no evidence of regrowth (Fig. 2B).

The ambiguity of this case presentation led us to consider the genetic bases for coexistent different mutations, that is, the autosomal recessive inheritance in SCD with an autosomal dominant inheritance of CCx. However, some individuals with CCx present with a sporadic type of inheritance or do not show an identifiable mutation of the PRKAR1A gene (a tumor suppressor gene). Researchers believe that additional and as yet unidentified genes may cause the disorder in these cases (genetic heterogeneity).^[19]^

While studies have shown that the mutations of the Protein Kinase CAMP-Dependent Type I Regulatory Subunit Alpha (PRKAR1A) gene (which is located on chromosome 17), are mainly responsible for CCx, there have been several reports of mutations on the short arm of chromosome 2 being involved as well.^[19]^ Interestingly, there are several other diseases that have symptoms similar to those of CCx,^[19]^ further emphasizing the need to explore the genetic basis for diseases that coexist with CCx.

On the basis of these findings, investigators should identify additional genes that could link both conditions together, especially as SCD is composed of various genotypes. Nearly all genetic studies of SCD have concentrated on this β-globin genotype found on chromosome 11. However, other genotypes are the result of compound heterozygosity for HbS and other hemoglobin variants, including HbC, HbE, HbD, and HbS-β thalassemia.^[20]^

## 3 Conclusions

This study is the first report of primary pigmented meningeal melanocytoma in CCx and of CCx with SCD. As meningeal melanocytomas are considered benign, complete surgical resection can be curative in most cases, and subtotal resection can also have a good outcome and prognosis. Altered gene mutations involved in variable morphogenesis and transcriptional regulation potentiate the further study of genes enriched in CCx, particularly those with coexisting SCD. The observation of coexistence of CCx and SCD in this case suggests the intriguing possibility of shared genetic contributions in both entities and provides opportunities for improved prognostic assessment and early therapeutic intervention in CCx patients.

## Acknowledgments

The authors would like to thank Dr. Abdulkader Marwah Mohammed, a pathology consultant, for her valuable assistance in obtaining the microscopic pathology images. This research did not receive any specific grant from funding agencies in the public, commercial, or not-for-profit sectors.

## Author contributions

**Investigation:** Abdulrahman Hamad Al-Abdulwahhab, AbdulAziz Mohammad Al-Sharydah, Sari Saleh Al-Suhibani, Hadeel Al-Shayji, Ibtihal Al-Saad, Wissam Al-Issawi.

**Resources:** Abdulrahman Hamad Al-Abdulwahhab, AbdulAziz Mohammad Al-Sharydah, Sari Saleh Al-Suhibani, Hadeel Al-Shayji, Ibtihal Al-Saad, Wissam Al-Issawi.

**Writing – original draft:** Abdulrahman Hamad Al-Abdulwahhab, AbdulAziz Mohammad Al-Sharydah, Sari Saleh Al-Suhibani, Hadeel Al-Shayji, Ibtihal Al-Saad, Wissam Al-Issawi.

**Writing – review & editing:** Abdulrahman Hamad Al-Abdulwahhab, AbdulAziz Mohammad Al-Sharydah, Sari Saleh Al-Suhibani, Hadeel Al-Shayji, Ibtihal Al-Saad, Wissam Al-Issawi.

## References

[1] Jellinger K Chou P Paulus W Kleihues P Cavanee WK Melanocytic lesions. International Agency for Research on Cancer, World Health Organization Classification of Tumours: Pathology and Genetics of Tumours of the Nervous System.. Lyon, France: 2000.

[2] Lee NK Lee JY Wang KC Primary atypical melanocytoma arising from the cavernous sinus in a child. Childs Nerv Syst 2015;31:1577–82. 25982184 10.1007/s00381-015-2741-3

[3] Brat DJ Perry A Louis DN Ohgaki H Wiestler OD Cavenee WK Melanocytic lesions. IARC, WHO Classification of Tumours of The Central Nervous System. Lyon: 2007.

[4] Girolami I Cima L Ghimenton C NRAS Q61K mutated diffuse leptomeningeal melanomatosis in an adult patient with a brief review of the so-called “forme fruste” of neurocutaneous melanosis. Brain Tumor Pathol 2018;35:217–23. 30145692 10.1007/s10014-018-0328-x

[5] Botticelli AR Villani M Angiari P Meningeal melanocytoma of Meckel's cave associated with ipsilateral Ota's nevus. Cancer 1983;51:2304–10. 6850510 10.1002/1097-0142(19830615)51:12<2304::aid-cncr2820511223>3.0.co;2-u

[6] Doglietto F Colosimo C Lauriola L Intracranial melanocytic meningeal tumours and melanosis oculi: case report and literature review. BMC Cancer 2012;12:220–6. 22672887 10.1186/1471-2407-12-220 PMC3489543

[7] Faro SH Koenigsberg RA Turtz AR Melanocytoma of the cavernous sinus: CT and MR findings. AJNR Am J Neuroradiol 1996;17:1087–90. 8791920 PMC8338603

[8] Lee JK Rho YJ Jeong DM Diagnostic clue of meningeal melanocytoma: case report and review of literature. Yonsei Med J 2017;58:467–70. 28120582 10.3349/ymj.2017.58.2.467 PMC5290031

[9] Gamoh S Tsuno T Akiyama H Intracranial meningeal melanocytoma diagnosed using an interdisciplinary approach: a case report and review of the literature. J Med Case Rep 2018;12:177–81. 29941032 10.1186/s13256-018-1725-9 PMC6020204

[10] Chen CJ Hsu YI Ho YS Intracranial meningeal melanocytoma: CT and MRI. Neuroradiology 1997;39:811–4. 9406208 10.1007/s002340050510

[11] Kurita H Segawa H Shin M Radiosurgery of meningeal melanocytoma. J Neurooncol 2000;46:57–61. 10896205 10.1023/a:1006335616839

[12] Pan H Wang H Fan Y Intracranial meningeal melanocytoma associated with nevus of Ota. J Clin Neurosci 2011;18:1548–50. 21924617 10.1016/j.jocn.2011.01.040

[13] Carney JA Gordon H Carpenter PC The complex of myxomas, spotty pigmentation, and endocrine overactivity. Medicine (Baltimore) 1985;64:270–83. 4010501 10.1097/00005792-198507000-00007

[14] Piercecchi-Marti MD Mohamed H Liprandi A Intracranial meningeal melanocytoma associated with ipsilateral nevus of Ota: case report. J Neurosurg 2002;96:619–23. 11883852 10.3171/jns.2002.96.3.0619

[15] Navas M Pascual JM Fraga J Intracranial intermediate-grade meningeal melanocytoma with increased cellular proliferative index: an illustrative case associated with a nevus of Ota. J Neurooncol 2009;95:105–15. 19449182 10.1007/s11060-009-9907-3

[16] Küsters-Vandevelde HV van Engen-van Grunsven IA Küsters B Improved discrimination of melanotic schwannoma from melanocytic lesions by combined morphological and GNAQ mutational analysis. Acta Neuropathol 2010;120:755–64. 20865267 10.1007/s00401-010-0749-z PMC2991233

[17] Rades D Schild SE Tatagiba M Therapy of meningeal melanocytomas. Cancer 2004;100:2442–7. 15160350 10.1002/cncr.20296

[18] Brat DJ Giannini C Scheithauer BW Primary melanocytic neoplasms of the central nervous system. Am J Surg Pathol 1999;23:745–54. 10403296 10.1097/00000478-199907000-00001

[19] Stratakis CA Carney JA Lin JP Carney complex, a familial multiple neoplasia and lentiginosis syndrome. Analysis of 11 kindreds and linkage to the short arm of chromosome 2. J Clin Invest 1996;97:699–705. 8609225 10.1172/JCI118467 PMC507106

[20] Habara A Steinberg MH Minireview: Genetic basis of heterogeneity and severity in sickle cell disease. Exp Biol Med (Maywood) 2016;241:689–96. 26936084 10.1177/1535370216636726 PMC4950383

